# Cancer Immunology: From Molecular Mechanisms to Therapeutic Opportunities

**DOI:** 10.3390/cells11030459

**Published:** 2022-01-28

**Authors:** Fabrizio Mattei, Carlos Alfaro, Yona Keisari

**Affiliations:** 1 Department of Oncology and Molecular Medicine, Istituto Superiore di Sanità, Viale Regina Elena 299, 00166 Rome, Italy; 2 Centre for Applied Medical Research, Universidad de Navarra, Av. Pio XII 55, 31008 Pamplona, Spain; 3 Department of Clinical Microbiology and Immunology, Sackler Faculty of Medicine, Tel Aviv University, Tel Aviv 6997801, Israel

## 1. Preface to the Special Issue

The gradual and more profound dissection of the molecular basis of cancer progression, carcinogenesis, and metastatic spread of cancer cells has led to more focused, effective, and targeted therapeutic approaches in the disparate types of solid and hematological tumors, particularly those with high ability to metastasize distant organs. Concurrently, identification of multiple pathways involved in cancer progression or, vice versa, in the eradication of tumors, accentuated the pivotal role of the immune response in this context.

A new era of immuno-oncology arose in the last 20 years, which revolutionized the concept of cancer progression as an event not merely depending on cancer cells alone, but rather associated with a struggle between the immune system and the developing tumor in the host. Cancer immunology is a growing field that investigates the complex mechanisms and interactions between immune and cancer cells, with the aim of identifying therapeutic targets for immunotherapy-based approaches.

The enormous and impressive results now reached in cancer research and therapy, were unthinkable without the valuable contribution of animal models to study the various types of tumors. Moreover, the advent of methodological approaches endowed with the ability to instantaneously investigate the gene profiling of cancer cells and immune systems, such as RNA microarrays profiling and single-cell RNA sequencing, led to an impressive collection of huge databases immuno-oncologists and bioinformaticians can rely on to deal with more optimal chemo-, radio- or immunotherapeutic strategies for virtually every type of cancer.

This issue presents a collection of reports and reviews on key scientific problems associated with all fields of immuno-oncology, from cancer research and clinical oncology to cancer therapy approaches. These reports can significantly contribute to paving the way to novel effective therapeutic interventions of significant benefit for cancer patients.

## 2. Special Issue “Cancer Immunology: From Molecular Mechanisms to Therapeutic Opportunities”

This Special Issue, composed of 15 research articles and 13 review reports, represents relevant knowledge in all the fields directly or indirectly involved in cancer immunology, cancer research, and therapeutic strategies of cancer, including combinatorial approaches to provide a more effective eradication of the tumors. These contributions have been presented by international experts in the field related to cancer research, cancer immunology, and cancer therapy.

Specifically, these contributions include studies on the implementation of already existing tumor mouse models or the generation of novel types of animal models to study rare types of cancers. Furthermore, there are interesting reports on radiation therapy, combination therapies, and articles on particularly aggressive types of neoplastic diseases. Other reports debate on the role of myeloid cells and inflammation pathways in some tumors, as well as the exploitation of immune checkpoint inhibitors in more targeted therapeutic approaches, with the aim to better optimize the efficacy of the immune system to fight cancers.

### 2.1. Use of Animal Models in Cancer Research

An interesting study is included that exploits an equine (*Equus caballus*) tumor model for papillomavirus-induced penile cancer, a rare tumor whose cancer microenvironment details are still under intense investigation. Here, Porcellato et al. studied the characteristics of the tumor immune microenvironment (TIME) in animals affected by equine penile cancer caused by the *Equus caballus* papillomavirus type 2 (EcPV-2). They found an increased infiltration of macrophages, Foxp3+ Treg cells, and neutrophils, paralleled to a decreased expression of *IFNG* and *IL2* mRNAs. In addition, they observed increased levels of neutrophils in EcPV-2high viral load samples. This particular TIME status significantly contributes to penile cancer progression. Such an equine model may furnish important knowledge to the investigations related to human penile cancers [1].

A study performed by Ciccolella et al. used mouse models to set up a sublingual administration of Type I Interferons (IFN-I) and demonstrated that such a particular type of cytokine administration achieved clear anticancer effects on melanoma and lymphoma during monotherapy and in combination with chemotherapy. When IFN-I was administered in a sublingual fashion, together with cisdiamminedichloroplatinum (II) (CDDP), in a B16.OVA tumor mouse model, a delayed melanoma growth associated with increased production of IFN- was observed, with clear antitumoral effects. Cyclophosphamide (CTX) does exert similar effects on an EG7.OVA lymphoma mouse model. Specifically, there was a strong delay in tumor growth, paralleled by increased levels of infiltrating CD3+ T lymphocytes. This contribution underlines how unusual but safe and effective IFN-I administration methods can be more beneficial for future optimization of chemo- or immunotherapeutic protocols for cancer patients [2].

These reports demonstrate the valuable use of animal models in particular situations where specific events or therapeutic treatments cannot be easily investigated in humans.

### 2.2. Role of Tumor Necrosis Factor Receptors in Tumors

Tumor necrosis factor receptors (TNFRs) have long been studied regarding their role in cancer. They can indeed activate cell death or induce the expression of factors involved in cellular differentiation and survival. There are two TNFRs—TNFR1 and TNFR2—using divergent pathways to direct the tumor fate. For these reasons, TNFRs are still under intense investigation to dissect their precise role in cancer under the different scenarios of cancer progression.

Rodriguez et al. investigated the role of TNFR1 using a B16.F1 melanoma mouse model and mice deficient for TFNR1 (TFNR1-KO mice). Here, they showed that the deficiency of this receptor (compared with WT mice) delays the B16.F1 melanoma growth in vivo and reduces the B16.F1 cell proliferation by decreasing the proliferating cell nuclear antigen (PCNA) expression in the tumor mass. In addition, melanoma-bearing TNFR1-KO mice showed defects in the development of angiogenesis in the B16 tumor microenvironment. Furthermore, the melanoma microenvironment derived from TNFR1-KO mice showed an increased infiltration and activation of CD8+ T lymphocytes with concomitant induction of CCL2, a chemokine recognized for its selective ability to attract T cells [3].

An interesting study provided novel insights on the role of TNFR2 in triple-negative breast cancer (TNBC). Here, Baram et al. identified and characterized two novel subsets of tumor-infiltrating lymphocyte (TIL), both expressing the TNFR2 receptor on their surface, which can play relevant roles during TNBC chemotherapy. TNFR2 is usually expressed in CD4+ T effector cells, but there are some identified subsets also expressing Foxp3, both in humans and mice. These CD4+TNFR2+Foxp3+ cells are Treg cells with potent immunosuppressive activity inside the tumor microenvironment. TNFR2 is also expressed in CD8+ T lymphocytes. The relevant findings of this report indicate that after chemotherapy, there was a marked decrease in CD4+TNFR+Focp3 TILs, with a concomitant increase in CD8+TNFR2+PD-1 TILs. Notably, this was associated with a decreased tumor mass, thus providing a positive impact on survival following chemotherapy. Finally, the authors demonstrated that the two novel TIL subsets (CD8+TNFR2+ and CD8+TNFR2+PD-1+) were present in TNBC patients before chemotherapy and were enriched after chemotherapy, with a parallel reduction in CD4+TNFR2+Foxp3+ Treg cells. Overall, these two CD8+ TILs expressing TNFR2 have clear beneficial effects on TNBC patients following chemotherapy [4].

These facts on the impact of TNFR1 and TNFR2 expression in the context of cancer progression provide new findings on the role of these two receptors in cancer and also delineate the importance of a better characterization of the immune contexture in the tumor microenvironment. Then, profiling and characterizing the infiltration of immune cells in primary tumors matters.

### 2.3. Myeloid Cells and Their Multifaceted Roles in Tumor Progression

Myeloid cells comprise disparate types of cell populations, all derived from the common myeloid progenitor. These cells have been recognized to exert both anti- or protumoral effects on cancer progression and metastatic spread, depending on exact tumor status and on how they interact with the other components of the host immune system.

In a report debating on the role of myeloid cells in the development of liver diseases, Sun et al. reviewed the impact of triggering receptors expressed on the myeloid cell (TREM) family, on such malignancies. This family indeed includes TREM1 and TREM2, important members involved in the control of inflammation in liver diseases. Accurate knowledge of the mechanisms by which these two members can modulate liver inflammation is of key relevance because they often develop in liver tumors. In hepatic tissue, TREM1 and TREM2 are usually expressed by sinusoidal endothelial cells, Kupffer cells, and hepatic stellate cells. In summary, this review denotes the importance of TREM1/2 in eliciting multiple pathways to control inflammation in the liver and thus the fate of cancer development. This scenario depends on the balance of TREM1 or TREM2 expression on the surface of Kupffer cells. Indeed, TREM1 elicits the homing of neutrophils and macrophages within the liver microenvironment, and this led to the induction of inflammation via the production of multiple pro-inflammatory cytokines. This scenario can potentially expose liver tissue to cancer development within the inflamed regions. On the other hand, the expression of TREM2 on Kupffer cells indeed contrasts the production of inflammatory cytokines and halts myeloid-cell-mediated tumor progression [5]. These data pave the way to novel ways of interactions between myeloid cells and tissue-resident cells to stimulate inflammatory processes during cancer development.

Continuing with the exposition, Huang et al. summarized the relationships between inflammatory cytokines and adhesion molecules inside the tumor microenvironment. Existing associations between these two types of molecules are of key relevance, both for the infiltration of myeloid cells responsible for the immune suppression into the tumor microenvironment and for the selection of common signaling pathways able to activate the proliferation of cancer cells. This review underlines the role of sialylation and glycosylation as key metabolic processes, which can establish the fate of the tumor by modulating the interactions of immunosuppressive myeloid cells, such as Tregs or MDSCs, with emphasis on gastrointestinal cancer [6].

In a clinical research report on basophils derived from ovarian cancer patients, Bax et al. display the responsiveness of these cells to the bacterial peptide formyl-methionyl-leucyl-phenylalanine (fMLP), anti-Fcε receptor I (FcεRI) antibody, or anti-IgE antibody. This basophil activation test (BAT) indicates that the majority of responders act by exploiting all the three factors, whereas only a minor fraction of basophils from cancer patients are able to specifically respond to one factor only. Furthermore, The authors exhaustively show how the detection and characterization of these basophils, derived from ovarian cancer cells by BAT, can represent a useful ex vivo tool to identify cancer patients with hypersensitivity to the elected chemotherapeutic agents or basophil-associated allergy. This will allow to readdress these patients in more effective therapeutic strategies as well as to predict the therapeutic outcomes with more reliability, avoiding the problems linked to possible allergy- or hypersensitivity-related issues [7].

Among particular myeloid cell populations whose role in the tumor microenvironment is poorly investigated are human lung macrophages (HLMs). An interesting report from Braile et al. stresses the role of thymic stromal lymphopoietin (TSLP) in orchestrating the action of HLMs in the tumor microenvironment. In this study, the authors offered evidence that peritumoral and intratumoral regions of lung cancer express TSLP receptor (TSLPR) and IL7Rα, which are the two subunits of the heterodimeric TSLP receptor. This renders lung cancer sensitive to the long-form of TSLP (lfTSLP), selectively released by HLMs. Exposing HLMs to lfTSLP stimulate these cells to produce proinflammatory and angiogenic factors (TNF-α), which can, in turn, promote lung cancer progression. This research indicates the importance of the TSLP system (via the selection of distinct TSLP isoforms) and underlines the ability of the lfTSLP, produced by HLMs, in directing the fate of lung cancer [8].

A paper investigating the role of M2-polarized macrophages in pancreatic cancer reported novel molecular pathways that sustain the polarization and promote tumor progression via eHSP90α. Here, CD91 binds to toll-like receptor-4 (TLR4) and activates two distinct pathways—IKKα/β-NFkB-IRF3 and JAK2/TYK2-STAT-3—via the IRAK4 and MyD88, respectively. Both contribute to the downregulation of the inflammatory cytokines TNF-α and IL-1β with a concomitant upregulation of several M2-related cytokines. The activation of these pathways ultimately leads to tumor progression via M2-polarized macrophages, maintained by eHSP90α [9].

The role of several myeloid cells can also be modulated by tumor-derived extracellular vesicles to redirect the cancer progression. An elegant study from Gassmann et al. indeed underlines the relevance of extracellular vesicles (EVs, identified by the surface expression of CD63 and CD81 exosome markers) released by tumor cells from Ewing’s sarcoma to impede dendritic cell (DC) maturation and functions. Here, the authors display that Ewing’s Sarcoma (EwS)-derived EVs induce proinflammatory stimuli in CD33+ and CD14+ myeloid cells, but also shift their maturation toward monocytic-derived DCs (moDCs). Notably, EwS EVs are capable to redirect CD33+ and CD14+ myeloid cell gene expression profiles toward inflammation and suppression of moDC maturation. Moreover, EwS-derived EVs inhibit the ability of moDCs to activate T lymphocytes, due to suppression of DC maturation. These findings underline novel key mechanistic roles on the EV-mediated inflammatory pathways for EwS [10].

This body of evidence suggests that disparate subsets of myeloid cells infiltrating the tumor microenvironment can orchestrate multiple spatiotemporal aspects of tumor progression and can represent optimal targets for future therapeutic strategies to cure cancer patients. Macrophages seem to play relevant roles in these scenarios.

### 2.4. Metastatic Expansion: New Mechanisms and Future Directions

In view of the fact that metastases are responsible for the death of at least 67% and up to 90% of cancer patients, depending on the tumor type, the development and expansion of metastases are among important aspects under intense investigation in cancer research. Dissecting the mechanisms at the basis of metastatic spread is a key prerequisite to optimizing chemo- and immunotherapeutic protocols for cancer patients.

By using a xenograft model of human melanoma brain metastasis (MBM) in nude mice, Moshe et al. focused on dissecting the disparate tumor-intrinsic, microenvironmental, and downstream factors involved in MBM development and its crosstalk with brain resident cells. Here, the authors investigated the reason for the heterogeneous responses to granulocyte-macrophage colony-stimulating factor (GM–CSF) displayed by melanoma patients. Indeed, GM–CSF is usually employed as an adjuvant in several clinical studies, with contrasting results. The findings presented in this report indicate that the interactions between the brain microenvironment and MBM cells, entering into the brain, are of relevance for the upregulation of GM–CSF in the metastatic regions of the brain. Thus, when GM–CSF is upregulated in the brain, it alters the gene expression profile of brain endothelial cells, which readapt to harbor metastatic cells. This cytokine is, therefore, detrimental in controlling the fate of melanoma metastatic cells within the brain microenvironment by generating brain regions similar to premetastatic niches. For example, GM–CSF has been shown to diminish the expression of Claudin-5 and Zonula-Occludens-1, two key adhesion proteins expressed in brain tissue. This suggests that GM–CSF may orchestrate the permeability of metastatic melanoma cells by acting on tight junction structures. On the other hand, GM–CSF expression can also be affected by the presence of proinflammatory cytokines (TNF-a and IL-1) in the brain tissue. These findings clearly suggest that the fate of MBM migrating in the brain is directed by an intricated cytokine network, where GM–CSF seems to play a key role and may also be responsible for the heterogeneity of GM–CSF response in clinical trials [11].

The use of a novel epigenetic model to categorize glioma patients based on their immune responsiveness was proposed by Polano et al. Here, a total of 573 cases of low-grade glioma (LGG) and glioblastoma were analyzed by an ad hoc machine learning algorithm, to automatically select and stratify glioma patients by comparing the immune response-related genes and the epigenetic pathways. This novel classification method can represent a valid smart bioinformatics tool for a better enrollment of brain cancer patients for therapeutic interventions [12].

An interesting review by Shurin et al. analyzed the role of the neuroimmune axis in the generation of metastasis in surgically removed tumors, providing evidence of how often excised tumors could present postoperative formation of metastasis. Notably, metastatic growth during the postoperative phase occurred both in mouse models and humans. This adverse event can arise in practically all types of tumors. However, the mechanisms behind such a metastatic regeneration are still unknown and represent an urgent matter to deal with. Recently, particular types of neurofilaments have been displayed within the tumor microenvironment of several cancers, thus suggesting that the modulation of the neuroimmune axis in the context of cancer progression can represent an important mechanism that could explain the frequent postoperative generation of metastatic foci. This report reviewed and debated the insurgence of a novel concept in which the immune system interacts with neuronal derivatives in the tumor microenvironment, defined as neuro-immuno-oncology. Dissecting the neuroimmune regulation during cancer progression can be a valid help to avoid the clinical adverse postoperative consequences during cancer excision, mainly the formation of postoperative metastasis [13].

Tumors developing in bone tissues constitute another type of neoplastic malignancy potentially exposed to metastasis formation. In this regard, the immuno-oncology of osteosarcomas and bone metastasis is currently an unmet clinical challenge and is still under intense debate. A review is presented in this Special Issue that deals with the introduction of the novel concept of osteo-immuno-oncology (OIO). This term defines all the direct and indirect relations between the immunity of bone tissues and its crosstalk with bone cancer development and metastasis formation. Notably, OIO includes all the specific therapeutic targets which can be adopted to contrast bone tumors and bone tissue metastatic expansion [14].

Cancer stem cells (CSCs) are multipotent cells able to self-renew and induce intratumoral heterogeneity, tumor progression metastatic spread, and drug resistance. Galassi et al. presented a review in which they debated on their immune-privileged status during all three phases of the immunoediting process (elimination, equilibrium, escape). Indeed, the downmodulation of relevant surface molecules involved in immune visibility represents a mechanism CSCs activate for immune escape. For example, a diminished expression of ULBP1, ULBP3, and MICA/MICB impedes NK cells to kill CSCs via NKKG2D. Similar mechanisms may also occur for other immune cells, such as macrophages and CD8+ T cells. Notably, CSCs are endowed with the capability to form niches within the TME or metastatic niches that sustain the metastasis formation. The deep knowledge of these and other mechanisms CSCs exploit to escape immunity is pivotal for the development of strategies aiming at targeting these cells to eradicate cancer. In this context, CSCs express a plethora of tumor-associated antigens (TAAs) on their surface, and most of them are still under intense investigation. Therapeutic strategies targeting some of these TAAs on CSCs can be promising approaches to overcome CSC-dependent drug resistance and tumor recurrence [15].

Hypoxia and acidity are key factors that allow tumor progression and are associated with poor clinical outcomes. Decoding the molecular mechanisms by which hypoxia and acidity orchestrate melanoma exosome (Mexo) release and molecular content is of great interest and represents a great challenge in the field of melanoma. Boussadia et al. aimed to examine melanoma development in its systemic context, represented by acidic and hypoxic conditions in the multifaceted interplay of tumor cells with stromal immune and non-immune cells. A precise knowledge of the Mexo content is also pivotal for the prevention of melanoma’s ability to metastasize distant organs. In addition, this review also debates the role of mesenchymal stem cells (MSCs) in the melanoma microenvironment, which can undergo phenotype activation and subsequent malignant transformation by Mexo. For these reasons, MSCs represent key cell subsets to deal with in the context of contrasting melanoma metastatic expansion. This review suggests that the study of MExo secreted in the melanoma microenvironment is a helpful strategy for the characterization of altered signals essential for tumor growth and metastatic spread, as well as for the investigation of new biomarkers [16].

Together, this set of inspiring findings can represent a relevant body of evidence useful to optimize the strategies aimed at contrasting the generation of metastasis during cancer progression or after postoperative excision of tumor tissues.

### 2.5. Radiotherapy and Tumor Ablation: First-Line Tools to Contrast Development of Tumors and Trigger the Immune Response

Tumor ablation can be defined as a minimally invasive technique to eradicate all viable malignant cells within a designated target volume through the application of energy or chemicals. Methods such as radiotherapy, chemical and biological ablation, photodynamic therapy, cryoablation, high-temperature ablation (radiofrequency, microwave, laser, and ultrasound), and electric-based ablation have been developed for focal malignancies.

These concepts were extensively reviewed by Korbelik et al., addressing the effectiveness of tumor ablation, with particular emphasis on interventional oncology. The authors specifically debated the use of the N-dihydrogalactochitosan (GC) family of molecules for the optimization of tumor ablation strategies, mainly thermal ablation and photodynamic therapy (PDT). Korbelik et al. illustrated whether and how tumor ablation could be properly combined with GC to a more effective abolition of the tumor mass. Indeed, the use of GC is of relevance in that it directly stimulates antigen-presenting cells to produce an effective Th1 response. Notably, GC also exerts a direct tumoricidal activity on tumor cells. The use of GC-based combination strategies would be a valid approach with particularly aggressive tumors or cancer patients with relapse [17].

In an exhaustive review, Keisari and Kelson evaluated the current strategies aimed at optimally combining radiotherapy (RT) with immunomanipulation to obtain a complete eradication of cancer. Here, the authors underlined the urgent need to combine the disparate RT strategies, mainly particle-based RT, with other approaches able to stimulate the immune system, such as immunoadjuvants, immune-suppressor cells inhibitors, or inhibition of immune checkpoints molecules. Such combination treatments may achieve the destruction of non-irradiated tumor foci through the activation of antitumor immunity, a phenomenon designated as “the abscopal effect”. These therapeutic strategies are motivated by the fact that chemotherapy or immunotherapy alone is often not effective in eradicating residual cancer cells or metastasis. Here, a description of proton and carbon ion RT, as well as RT approaches based on the emission of alpha or photon particles, was reviewed in terms of application for tumor abolition. Keisari and Kelson also discussed how the abscopal effect can explain the central role of RT in the eradication of cancer cells from the body. Notably, the use of particle RT was extensively studied on different tumor mouse models to identify the precise effect on the immune system. For example, carbon ion and photon RT were employed in an LM-8 advanced osteosarcoma mouse model in combination with immune checkpoint inhibitors, which was demonstrated as effective in the reduction in metastasis. Notably, examination of the abscopal tumors from animals revealed an intense infiltration of CD8+ effector T cells [18].

A clinical report from Boustani et al. evaluated the effects of neoadjuvant photon RT and RT fractionation on the programmed death-ligand 1 (PD-L1) expression in locally advanced rectal cancer (LARC) patients. The authors collected data from biopsies and surgical samples from a cohort of 74 LARC patients. They detected an augmentation of PD-L1 expression in LARC patients subjected to neoadjuvant RT. The authors also found no correlation between RT fractionation and PD-L1 levels. However, a heterogeneous expression of PD-L1 was found in this cohort, with weak to very high levels. Conclusively, this report strongly suggested the importance of PD-L1 expression in LARC patients, which could, in some cases, explain the resistance of LARC patients to RT treatments [19].

Another clinical study from Taussky et al. investigated the impact of brachytherapy RT (a form of RT where a sealed radiation source is placed inside or next to the area requiring treatment) on a cohort of prostate cancer (PCa) patients and proposed that several blood inflammatory factors could be employed as predictive markers for PCa. Specifically, the authors demonstrated that neutrophil/lymphocyte ratio (NLR) and platelet/lymphocyte ratio (PLR) could be used to establish whether or not PCa patients can receive successive therapeutic strategies such as hormonal treatment or a combined external beam RT, a form of RT that directs high-energy X-ray beams at the tumor mass from outside the body. [20].

Hader et al. demonstrated the usefulness of a combined therapeutic approach exploiting RT and hyperthermia (HT) as an alternative energy source. Indeed, HT plus RT currently represents a promising RT-based combined approach to cure immunogenic tumors. However, the impact on immune cells and the mechanism behind this novel approach are still not fully identified. Furthermore, the exact radiative parameters to be used for an effective HT therapy are still under investigation. Here, the authors focused on the exploration of effective HT-plus-RT combinations that displayed the most effective activation of immune cells with concomitant induction of tumor cell death. Interestingly, Hader et al. designed a fully operative in vitro system to apply HT, allowing them to compare cell death induction with the expression of immune checkpoint factors. In these settings, the authors were able to study in vitro the HT-plus-RT combination therapy on B16 melanoma cells and human breast cancer cell lines (MDA-MB-231 and MCF-7). This report can establish future optimal HT-plus-RT strategies to be applied in vivo in tumor mouse models, which can then be translated into effective clinical trials for cancer patients [21].

An interesting study from Zhu et al. applies a non-invasive ablation approach, called “high-intensity, focused ultrasound” (HIFU) to axillary lymph nodes (ALNs) metastasized by breast cancer cells. HIFU is a solid cancer treatment that uses the energy of highly focused ultrasound to pinpoint, heat-and-kill cancer cells. Unlike radiation and surgery, HIFU is a non-invasive procedure that leaves healthy tissue unharmed. Upon HIFU, the authors investigated the immune response efficacy and the ability of HIFU to induce the secretion of several cytotoxic factors (FasL, Perforin, and GranzymeB in ALNs). This study revealed that HIFU stimulates an effective immune response by infiltration of key immune cell subsets, such as CD4+ T and CD57+ T cells, with a concomitant secretion of cytotoxic factors that contrast the further diffusion of metastatic breast cancer cells present in the ALNs [22].

From these reports emerges the need for combination treatments that will build upon better optimization of in situ ablation-based therapeutic strategies, to increase the benefit for cancer patients on the one hand, and by the repurposing of new immune modalities such as checkpoint inhibitors, new adjuvants, and more effective immune suppressor cell inhibition, on the other.

### 2.6. Immune Checkpoint Blockade in Cancer Therapy: Pros and Cons

Immune checkpoint inhibitors (ICIs) are widely used in therapeutic strategies against cancer, as they can overcome resistance problems due to the expression of those molecules that can suppress the immune responses against multiple types of tumors.

Gascón et al. extensively reviewed whether and how circulating lymphoid cells can impact the treatment efficacy of ICIs in lung cancer. In this context, they compared the role of lymphoid cells present in the lung tumor microenvironment with respect to circulating ones. By analyzing relevant lines of experimental evidence in scientific literature, Gascón et al. attempted to demonstrate whether circulating and lung cancer infiltrating lymphoid cells can be used as reliable predictive biomarkers for lung cancer patients. They analyzed a significant set of clinical studies making use of ICIs in non-small cell lung cancer (NSCLC), an aggressive and often resistant form of lung tumor. Although circulating cells have already been extensively used as cell biomarkers for ICIs’ responsiveness, much should be carried out to better define the biomarkers for a more affordable prediction of NSCLC responders or non-responder. The comparison of immune cells infiltrating the lung tumor microenvironment with those circulating in the blood can constitute a better modality to predictively select ICI responders and non-responders in NSCLC patients [23].

There are certain situations where the expression of ICIs can provoke serious collateral effects in cancer patients. PD-1 and PD-L1 blockade with monoclonal antibodies (nivolumab and pembrolizumab for PD1; atezolizumab, avelumab, and durvalumab for PD-L1) now represent an effective treatment for various aggressive cancers (NSCLC, melanoma, head-and-neck cancer, kidney cancer) when combined with radiotherapy or chemotherapy. However, Correale et al. provided evidence that severe collateral effects can often occur during the course of therapy, which can seriously compromise the clinical follow-up of cancer patients and then the success of ICI-based chemo- or radiotherapy. These immune-mediated adverse effects are grouped as immune-mediated adverse events (irAEs), in that they are always directly or indirectly initiated by unpredictable dysfunctions of the immune system. Therefore, the discovery of markers predictive of such an irAEs is a current urgent need that can help to optimize ICI-based cancer therapy in those patients particularly affected by these effects. Correale et al. observed that ICI-related pneumonitis (IRP) constitutes one of the most common irAEs and often occurs in NSCLC patients who received anti-CTLA4 and PD-1/PD-L1 ICIs. ICI-treated melanoma patients can also be exposed to IRP but with a lesser severity compared to NSCLC ones. The urgency of IRP prediction for NSCLC patients under ICI therapy mainly relies on the fact that symptoms can evolve to irreversible damages such as hypoxia and cyanosis, leading to respiratory failure. By carrying out a multicenter, retrospective analysis of 256 cancer patients who received PD-1/PD-L1 blockade, the authors evinced that the risk of developing IRP is associated with the germinal expression of distinct class I and class II human leukocyte antigen (HLA) alleles. These findings are also of key relevance to prevent the exposure of ICI-treated NSCLC cancer patients to the emerging severe acute respiratory syndrome coronavirus 2 (SARS-CoV-2)-related pneumonitis [24].

### 2.7. Other Therapeutic Approaches for Cancer Abolition

A review from Magri et al. reported a potential role for the tumor-associated antigen xCT with a mutant form of the p53 protein for effective combination therapies for cancer. xCT is a newly identified antigen expressed on cancer cell surfaces and has also been found highly expressed on cancer stem cells. The expression of xCT on cancer cells protects them from ferroptosis and oxidative stress. It is then conceivable that xCT can be exploited for the generation of novel immunotargeting strategies. On the other hand, mutant p53 protein is endowed with the capability to induce a transcriptional downregulation of xCT with concomitant induction of ferroptosis. In this review the authors described the direct and indirect pathways between p53 and xCT, to be employed for new combination cancer therapies based on the use of mutant forms of p53 together with xCT [25].

Braile et al. summarized some peculiar therapeutic features of the *Cannabis sativa* related to cancer. The endocannabinoid molecules and synthetic cannabinoids associated with this plant share two subtypes of G-coupled proteins (CB1 and CB2), which are exploited by cannabinoids to regulate several aspects related to cancer. These receptors are expressed on several immune cell populations, and conceivably, they modulate a variety of functions of innate and adaptive immune systems. The full characterization of the human cannabinoid receptor pathways has been only recently completed and is opening the way to future anticancer therapeutic approaches targeting this receptor, based on the specific design of CB1 and CB2 agonists [26].

Another example where natural compounds can be used to fight cancer comes from a report describing the role of natural chemicals of marine origin. Here, Sansone et al. elucidated why these compounds, well recognized as potent inducers of apoptotic cell death in a variety of tumors, can represent potent natural factors to be used in cancer therapy, with extremely limited toxicity [27].

Large granular lymphocytic leukemia (LGLL) is a particular type of hematologic malignancy whose main features are the proliferation of NK cells or cytotoxic T-large granular lymphocytes. Moreover, the most important clinical parameter characterizing this tumor is the presence of marked neutropenia. Calabretto et al. debate the mechanisms involved in the development of LGLL-associated neutropenia, including the role of the miR-146b in these processes. LGLL is a heterogeneous neoplastic disease in that it spaces between several pathogenetic profiles, including autoimmune disorders and bone marrow (BM) failure syndromes. LGLL-associated neutropenia can be hypothetically dependent on the modulation of Fas-FasL. In this process, mir-R-146b does control the FasL mRNA expression via the human antigen R, an RNA-binding protein and an essential FasL mRNA stabilizer. Conclusively, this review provides bodies of evidence for the possible use of miR-146b as a therapeutic target to redirect the LGLL-associated neutropenia [28].

## 3. Conclusive Remarks

This Special Issue is composed of 15 research articles and 13 reviews, spanning several central topics related to cancer research, cancer immunology, and new therapeutic strategies to cure disparate types of cancer. We hope our Special Issue will inspire the immuno-oncology scientific community to provide novel and innovative ideas to boost the endless battle against cancer.

## Data Availability

Not applicable.
